# Trends and factors influencing the mental health of college students in the post-pandemic: four consecutive cross-sectional surveys

**DOI:** 10.3389/fpsyg.2024.1387983

**Published:** 2024-07-17

**Authors:** Yinhai Chen, Xiong Ke, Jinfeng Liu, Jun Du, Jiali Zhang, Xuan Jiang, Tong Zhou, Xiao Xiao

**Affiliations:** ^1^Primary Health Care Research Centre, North Sichuan Medical College, Nanchong, China; ^2^Mianyang Central Hospital, Mianyang, China; ^3^Department of Foreign Languages and Cultures, North Sichuan Medical College, Nanchong, China; ^4^North Sichuan Medical College, Nanchong, China; ^5^Central People’s Hospital of Zhanjiang, Zhanjiang, China

**Keywords:** COVID-19, mental health, suicidal thoughts, PTSD, depression, anxiety, cross-sectional study

## Abstract

**Background:**

The long-term impact of COVID-19 on the mental health and well-being of college students, specifically trends over time after full removal of COVID-19 restrictions, has not been well-studied.

**Methods:**

Four consecutive cross-sectional surveys were conducted in December 2022 (*N* = 689), March 2023 (*N* = 456), June 2023 (*N* = 300), and November 2023 (*N* = 601) at a university in Sichuan Province, China.

**Results:**

The proportion of students with COVID-19 panic decreased from 95.1 to 77.3% (*p* < 0.001). The prevalence of moderate anxiety and above decreased from 18 to 13.6% (*p* < 0.001), and the prevalence of moderate and above depression decreased from 33.1 to 28.1% (*p* < 0.001), while the prevalence of post-traumatic stress disorder (PTSD) increased from 21.5 to 29.6% (*p* < 0.005). Further, the proportion of suicidal thoughts increased from 7.7 to 14.8% (*p* < 0.001). Suicidal thoughts and self-injuries were significantly associated with COVID-19 panic, depression, anxiety, and PTSD. Students who reported being in close contact with COVID-19 patients in the past were more likely to develop PTSD. Further, COVID-19-induced panic was a risk factor for self-injury.

**Conclusion:**

One year after the COVID-19 pandemic, the overall mental health of college students was not optimal. Hence, we can conclude that the long-term impacts of COVID-19 on the mental health of college students may have already occurred. To mitigate this impact and prepare for the next major public health event, strengthening college students’ mental health curricula and promoting healthy behaviors among college students should be a priority for universities and education authorities.

## 1 Introduction

COVID-19 was first detected in Wuhan, China, in December 2019 and declared a pandemic by the World Health Organization in March 2020 (Habas et al., 2020). As of March 2023, 676,609,955 people have been infected with COVID-19, including 6,881,955 deaths (Cheng et al., 2023). Throughout the pandemic, four infection peaks were observed in China: December 2019, May 2020, December 2021, and November 2022. During these periods, the Chinese government adopted a “dynamic zero COVID-19” strategy, which refers to rapid intervention in cases of infection, to ensure the population’s safety (Cheng et al., 2023). As the replicability and pathogenicity of Omicron significantly decreased (Shuai et al., 2022; Suzuki et al., 2022), it was found that mild and asymptomatic infections accounted for the vast majority of confirmed patients, resulting in a significant increase in virus transmission. Therefore, since December 7, 2022, The State Council has issued the “New Ten” prevention and control optimization measures for COVID-19 (Huang et al., 2023), which refers to 10 new measures to optimize the prevention and control of the epidemic. Since that time, there are no more isolation measures taken for COVID-19 positive patients, no more mandatory nucleic acid testing across the country, and everyone has free access to public places.

During a massive public health event like the COVID-19 pandemic, the mental health of college students requires special attention, as these individuals are in an important period of mental transformation, and their mental development is still incomplete (Lattie et al., 2019). According to research, about half of all mental disorders first begin in the mid-adolescence (Lipson et al., 2022). Unfortunately, the pandemic completely upended the daily lives of college students (Ratner et al., 2022). The vast majority of schools switched from in-person to online teaching, with a 3 year lockdown leaving students unable to move freely around campus and keeping them confined to their dormitories or homes (Branquinho et al., 2022; Qin et al., 2023). Moreover, students’ daily life and social interaction were affected. For students in dormitories, staff could only deliver meals and fruits to the downstairs of the dormitory, which were then delivered to them by other staff, and they could only communicate with their families and friends by mobile phone. Furthermore, students at home were not allowed to go to school or any other recreational venues. Since the relaxation of control measures, many college students have contracted the disease (Qin et al., 2023; Xiao et al., 2023). Further, the prolonged epidemic life of COVID-19 and the resulting state of elevated panic have taken a tremendous toll on students’ mental health and well-being (Cao et al., 2020; Tasso et al., 2021).

The major mental health problems reported among young people include depression and anxiety, as well as suicidal thoughts and behavior (Blader, 2018; Lattie et al., 2019). Note that the mental health level of college students before the pandemic was very different from that after the pandemic. One study from China found that the prevalence of depression and anxiety in pre-pandemic college students was 9.4 and 7.5%, respectively (Zhang et al., 2020). A pre-pandemic study based on 1,236 college students found that the prevalence of anxiety was 20.1% for women and 8.9% for men (Lee et al., 2016). Additionally, results from the World Health Organization’s World Mental Health Survey show that the 12 month prevalence of suicidal thoughts and behaviors among college students was 1.9% (Mortier et al., 2018).

However, a meta-analysis by Chang et al. (2021) showed that the prevalence of anxiety symptoms among college students during the pandemic was 31% and that the prevalence of depressive symptoms was 34% (Chang et al., 2021). A study of college students in the United States reported that the COVID-19 pandemic was associated with alarming rates of anxiety, depression, and suicidal thoughts (38.48, 48.14, and 18.04%, respectively) (Wang et al., 2020). Furthermore, a study in China showed that COVID-19 was an independent risk factor for anxiety (Cao et al., 2020). Studies have also shown that throughout 2020, cases of major depressive disorder increased by 27.6% globally, while cases of anxiety disorder increased by 25.6% (“Global prevalence and burden of depressive and anxiety disorders in 204 countries and territories in 2020 due to the COVID-19 pandemic,” Santomauro et al., 2021). Similarly, research by Taheri et al. showed that the COVID-19 pandemic had an impact on the mental health of athletes (Taheri et al., 2023a, 2023b). Another meta-analysis found that the overall prevalence of suicidal ideation during the pandemic was 12.1% higher than pre-pandemic rates (Farooq et al., 2021). Studies examining incidence rates of PTSD following the pandemic produced varied results; however, a recent meta-analysis of PTSD among medical students reported a prevalence of 34% (Peng et al., 2023). In light of these results, we can conclude that the COVID-19 pandemic has had a profound impact on the mental health of college students (Son et al., 2020; Wang et al., 2020; Mota et al., 2021; Tasso et al., 2021; Mosleh et al., 2022; Nuryana et al., 2022; Qin et al., 2023; Xiao et al., 2023).

However, it is important to note that most of these studies were short-term surveys and were conducted during the COVID-19 outbreak. Depression, anxiety, and PTSD symptoms typically exhibit a certain latency period. The incubation period for PTSD can range from weeks to 6 months or more (Chamaa et al., 2021). Therefore, it is likely that evidence regarding the long-term effects of COVID-19 on the mental health and well-being of college students remains limited, especially for trends over time in the mental health of college students after full removal of COVID-19 restrictions.

To fill this gap, four consecutive cross-sectional surveys throughout the year were conducted to study panic, depression, anxiety, PTSD, and suicidal thoughts among Chinese college students during the COVID-19 pandemic and within 1 year of the full reversal of lockdown restrictions. Specifically, this study had two aims: (1) to determine the trend over time of college students’ mental health, COVID-19 panic, and infection after the full removal of COVID-19 restrictions; and (2) to explore the influencing factors of mental health and COVID-19 panic among college students after the full removal of COVID-19 restrictions.

## 2 Methods

### 2.1 Study design and population

Four cross-sectional surveys were conducted: the first from December 14 to 20, 2022 (T1, just prior to the removal of COVID-19 restrictions), the second from March 1 to 7, 2023 (T2, 2 months after the removal of COVID-19 restrictions), the third from June 1 to 7, 2023 (T3, 5 months after the removal of COVID-19 restrictions), and the fourth from November 25 to 30, 2023 (T4, 10 months after the removal of COVID-19 restrictions). These specific time points were selected to capture immediate, short-term, and longer-term impacts of the removal of COVID-19 restrictions. The inclusion criteria were as follows: (1) age ≥ 18 years, (2) resident college student, and (3) no mental illness. The study sample was randomly recruited. All four surveys were conducted at a university in Sichuan Province, China. Five researchers randomly distributed paper questionnaires to different classes for the first three surveys. All participants agreed to participate in the survey, and the researchers were responsible for ensuring that the questionnaires were filled out completely. In both the second and third surveys, we made an effort to recall the students who had participated in the first study. Unfortunately, since our study was conducted at the beginning of the full opening up after the pandemic, almost all students had been infected the COVID-19, so only a small number of students were recalled by us to complete the follow-up survey. To ensure that the sample size of our study was large enough, we did our best to recruit new students in the second and third surveys. However, this led to a gradual reduction in our sample size. As time passed, many students went to different hospitals or companies to participate in clinical practice, and it was difficult to continue to issue paper questionnaires. Therefore, in the fourth survey, we used an online questionnaire platform to collect data, which increased the number of participants in our fourth survey. To ensure that the data of the fourth online questionnaire were as reliable as those of the previous three paper questionnaires, we used reverse questions in the questionnaire and screened the questionnaires of each participant individually. Questionnaires that were filled out in less than 180 s were regarded as unqualified. The same IP address could only be used once to complete a questionnaire, and all fields were set as mandatory. The same questionnaire was used for all four surveys, and all participants received a reward of 5 RMB after the survey was completed (students completing the fourth survey would not receive a reward if they took less than 180 s to complete the questionnaire). We set a confidence coefficient *Z* = 1.96, with an expected incidence of *p* = 0.2 and allowable error *d* = 0.05 within the 95% confidence interval. We calculated the required sample size (*n*) for each of the four surveys to be 246; considering that there could have been a sample loss of 10–20% after data collection was completed, we set the final sample size for four surveys to *n* = 270–295. The study was approved by the Ethics Committee of North Sichuan Medical College (ID No. 2023004).

### 2.2 Measures

#### 2.2.1 Demographic variables and situational factors

Collected demographic information included sex, grade, relationship with mother and relationship with father. Other information included whether the individual was infected with COVID-19, whether they had close contact with a COVID-19 patient, and whether they were worried about contracting COVID-19.

#### 2.2.2 Assessment scale

##### 2.2.2.1 Chinese version of fear of COVID-19 scale

The FCV-19S-C, developed by Ahorsu et al. in 2020, was sinicized and cross-culturally adapted to test fears of the COVID-19 pandemic (Ahorsu et al., 2022). The scale includes a total of seven items, each graded on a five-point Likert scale, from 1 (*strongly disagree*) to 5 (*strongly agree*). Higher scores indicate a higher level of fear, with a score greater than 16 considered COVID-19 panic. Cronbach’s alpha for this scale was 0.88.

##### 2.2.2.2 Patient health questionnaire 9

The PHQ-9 is internationally considered as the most reliable screening tool for depression, developed by Levis et al. (Levis et al., 2020; Costantini et al., 2021). This scale consists of nine self-rated items, each rated on a four-point Likert scale, ranging from 0 (*not at all*) to 3 (*nearly every day*) (Kroenke et al., 2001). A score of 0–4 is classified as normal, a score of 5–9 is classified as mild depression, a score of 10–14 is classified as moderate depression, a score of 15–19 is classified as moderate to severe depression, and a score of 20–27 is classified as severe depression. Cronbach’s alpha for this scale was 0.87.

##### 2.2.2.3 7-tiem generalized anxiety disorder scale

The GAD-7 was first developed by Spitzer et al., and it is widely used because of its simplicity and reliability (Spitzer et al., 2006; Plummer et al., 2016). The scale consists of seven items on a 21-point scale, with scores of 0–4 classified as no anxiety, 5–9 classified as mild anxiety, 10–14 classified as moderate anxiety, and 15–21 classified as severe anxiety. Cronbach’s alpha for this scale was 0.91.

##### 2.2.2.4 PTSD checklist-civilian version

The PCL-C measuring PTSD in civilians consists of three symptom groups: repeated experience symptoms (items 1–5), avoidance symptoms (items 6–12), and alertness symptoms (items 13–17) (Jing et al., 2022). Each item is rated on a five-point Likert scale (1 = *not at all*, 2 = *somewhat*, 3 = *moderate*, 4 = *considerable*, and 5 = *extreme*), with scores ranging from 17 to 85. A score of 17–37 points is classified as “no obvious PTSD symptoms,” 38–49 points is classified as “some PTSD symptoms,” and 50–85 is classified as “more obvious PTSD symptoms.” Cronbach’s alpha for this scale was 0.93.

#### 2.2.3 Other variables

Suicidal thoughts and self-harming behaviors were collected through two items: whether the respondent had experienced suicidal thoughts in the past and whether they had intentionally harmed themselves in the past.

#### 2.2.4 Statistical analyses

SPSS 27.0 was used for statistical analysis of data. Qualitative variables are described by frequency distribution and percentage, while quantitative variables are described by mean and standard deviation. To achieve the first major goal of the study—to explore the trends in students’ mental health, COVID-19 panic, and infection after the pandemic over time—we used the Wilcoxon signed rank to compare the changes in depression, anxiety, PTSD, suicidal thoughts, COVID-19 panic, and infection at four stages. Before regression analysis, a univariate analysis was performed for the FCV-19S-C, PHQ-9, AD-7, and PCL-C scales, and an independent sample *t*-test was used for variables with binary independent variables. For independent variables of three categories or more, ANOVA was used. Then, all the variables were included in the model for binary logistic regression analysis to control confounding factors (Rassolnia and Nobari, 2024) and achieve the second aim. The odds ratio (OR) and its 95% confidence interval (CI) were calculated as estimates of correlation. All *p*-values were two-sided, and a *p* < 0.05 was considered statistically significant.

## 3 Results

### 3.1 Participants and characteristics

A total of 689 college students participated in T1, 456 in T2, 300 in T3, and 601 in T4. The pass rate of the previous three questionnaires was 100%. Paper versions of the questionnaire were administered and collected on site. T4 was administered via an online platform, with a total of 615 college students participating. Among these, 14 questionnaires were excluded because the filling time was less than 180 s, resulting in 601 surveys analyzed (effective rate of 97%). As the survey sites were medical colleges, nursing majors represented the highest proportion of participants; thus, the proportion of female respondents was elevated. In the first three surveys, most of the students were freshmen and sophomores. Due to the large time span of this survey, we recalled them as much as possible during the fourth survey. Specific general information and details are shown in Table 1.

**Table 1:** Baseline characteristics of participants in four surveys.

	T1		T2		T3		T4	
	*N*	%	*N*	%	*N*	%	*N*	%
Total	689		456		300		601	
Gender								
Male	138	20.0	76.0	16.7	40.0	13.3	188	31.3
Female	551	80.0	380	83.3	260	86.7	413	68.7
Grade								
Freshman	394	57.2	235	51.5	189	63.0	139	23.1
Sophomore	282	40.9	207	45.4	110	36.7	212	35.3
Junior	13	1.9	14	3.1	1	0.3	200	33.3
Senior							50	8.3
Relationship with mother								
Poor	9	1.3	9	2.0	5	1.7	7	1.2
Better	125	18.1	73	16.0	39	13.0	128	21.3
Good	555	80.6	374	82.0	256	85.3	466	77.5
Relationship with father								
Poor	8	1.2	7	1.6	4	1.3	19	3.2
Better	175	25.4	100	21.9	65	21.7	187	31.1
Good	506	73.4	349	76.5	231	77.0	395	65.7
Suicidal thoughts								
No	636	92.3	418	91.7	281	93.7	512	85.2
Yes	53	7.7	38	8.3	19	6.3	89	14.8
Self-harm								
No	637	92.5	435	95.4	285	95.0	525	87.4
Yes	52	7.5	21	4.6	15	5.0	76	12.6
Past infection with COVID-19								
No	631	91.6	103	22.6	67	22.3	132	22.0
Yes	58	8.4	353	77.4	233	77.7	469	78.0
Close contact with COVID-19 patients								
No	101	14.7	237	52.0	168	56.0	143	23.8
Yes	588	85.3	219	48.0	132	44.0	458	76.2
Concern about COVID-19 infection								
No	182	26.4	198	43.4	127	42.3	327	54.4
Yes	507	73.6	258	56.6	173	57.7	274	45.6

### 3.2 Trends over time

**Figure 1:**
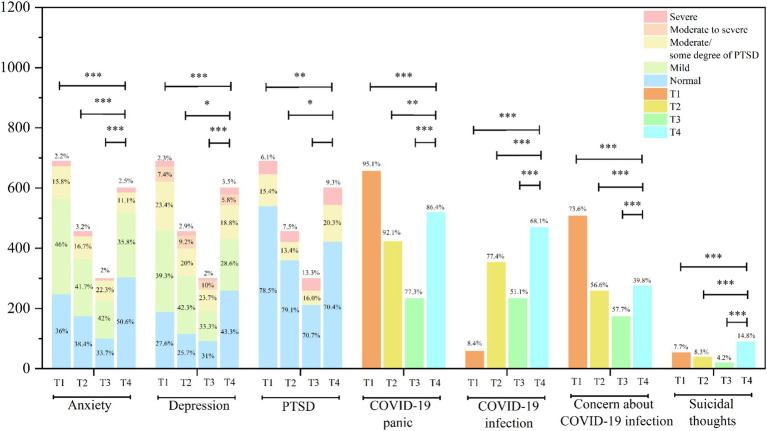
Time trends in anxiety, depression, PTSD, suicidal thoughts, and COVID-19 panic and infection. The length of each line represents the change in trend over the corresponding two time periods, and the stars above the lines represent the significance of the change, PTSD, post-traumatic stress disorder, *p < 0.05, **p < 0.005, and ***p < 0.001, the test results are presented at point 3.2.

We found that, at T1 (the period just prior to the removal of restrictions) and T2 (just after the removal of restrictions), students exhibited a high degree of panic about COVID-19, 95.1 and 92.1%, respectively. With the passage of time, this degree gradually decreased, with response at T3 and T4 showing that students’ fear of COVID-19 had dropped to 77.3 and 86.4%, respectively. These changes were statistically significant (*p* < 0.001). Similarly, the prevalence of moderate anxiety and above decreased from 18% in T1 to 13.6% at T4 (*p* < 0.001). The prevalence of moderate depression and above decreased from 33.1% in T1 to 27.1% at T4 (*p* < 0.001). Concern about COVID-19 infection decreased from 73.6% in T1 to 39.8% at T4 (*p* < 0.001). Rates of PTSD and suicidal thoughts did not decline. The prevalence of PTSD increased from 21.5% in T1 to 29.6% at T4 (*p* < 0.001) and suicidal thoughts also increased from 7.7% at T1 to 14.8% at T4 (*p* < 0.001), possibly owing to the months-long, or longer, incubation period for PTSD. Over time, the symptoms of PTSD gradually appeared and influenced the students’ suicidal thoughts. Moreover, we found that within 2 months after full removal of restrictions, the proportion of students infected with COVID-19 increased rapidly from 8.4 to 77.4% (*p* < 0.001; see Figure 1 for details).

### 3.3 Single-factor analysis of COVID-19 panic, depression, anxiety, and PTSD

We found that the weaker the relationship with parents, the higher the scores for depression, anxiety, and PTSD in respondents (all *p* < 0.001). Students with suicidal thoughts and self-harm had significantly higher scores for depression, anxiety, and PTSD (all *p* < 0.001). Students who had close contact with COVID-19 patients scored higher on the COVID-19 panic scale (*p* < 0.001; see Table 2).

**Table 2:** Single-factor analysis of COVID-19 panic, depression, anxiety and PTSD at T4.

	*N* (%)		COVID-19 panic	Depression	Anxiety	PTSD
Gender		*p*	0.054	0.235	0.331	0.716
Male	188 (31.3)		3.31 ± 0.57	0.80 ± 0.58	0.73 ± 0.64	1.97 ± 0.68
Female	413 (68.7)		3.39 ± 0.47	0.86 ± 0.55	0.79 ± 0.65	1.95 ± 0.66
Grade		*p*	0.007*	0.206	0.089	0.183
Freshman	139 (23.1)		3.40 ± 0.53	0.85 ± 0.53	0.77 ± 0.65	2.00 ± 0.73
Sophomore	212 (35.3)		3.39 ± 0.46	0.89 ± 0.60	0.83 ± 0.69	2.01 ± 0.69
Junior	200 (33.3)		3.38 ± 0.45	0.78 ± 0.52	0.69 ± 0.59	1.88 ± 0.57
Senior	50 (8.3)		3.09 ± 0.73	0.87 ± 0.53	0.89 ± 0.67	1.95 ± 0.66
Relationship with mother		*p*	0.787	<0.001***	<0.001***	<0.001***
Poor	7 (1.2)		3.49 ± 0.53	1.21 ± 0.64	1.27 ± 0.77	2.48 ± 0.71
Better	128 (21.3)		3.35 ± 0.46	1.07 ± 0.64	0.94 ± 0.74	2.18 ± 0.75
Good	466 (77.5)		3.37 ± 0.52	0.77 ± 0.51	0.72 ± 0.61	1.89 ± 0.62
Relationship with father		*p*	0.250	<0.001***	<0.001***	<0.001***
Poor	19 (3.2)		3.56 ± 0.47	1.27 ± 0.60	1.52 ± 0.81	2.62 ± 0.77
Better	187 (31.1)		3.35 ± 0.47	0.94 ± 0.58	0.84 ± 0.69	2.08 ± 0.69
Good	395 (65.7)		3.36 ± 0.53	0.77 ± 0.53	0.71 ± 0.59	1.87 ± 0.61
Suicidal thoughts		*p*	0.765	<0.001***	<0.001***	<0.001***
No	529 (88)		3.37 ± 0.50	0.76 ± 0.50	0.69 ± 0.59	1.88 ± 0.60
Yes	72 (12)		3.35 ± 0.59	1.40 ± 0.63	1.36 ± 0.76	2.57 ± 0.77
Self-harm		*p*	0.009*	<0.001**	<0.001**	<0.001**
No	525 (87.4)		3.35 ± 0.63	0.78 ± 0.51	0.70 ± 0.59	1.88 ± 0.60
Yes	76 (12.6)		3.51 ± 0.54	1.27 ± 0.65	1.27 ± 0.79	2.46 ± 0.81
Past infection with COVID-19		*p*	0.208	0.202	0.139	0.215
No	132 (22)		3.42 ± 0.45	0.79 ± 0.56	0.70 ± 0.63	1.89 ± 0.65
Yes	469 (78)		3.35 ± 0.52	0.86 ± 0.55	0.79 ± 0.65	1.98 ± 0.66
Close contact with COVID-19 patients		*p*	0.002**	0.030*	0.019*	0.005*
No	143 (23.8)		3.48 ± 0.47	0.75 ± 0.54	0.66 ± 0.66	1.82 ± 0.60
Yes	458 (76.2)		3.33 ± 0.51	0.87 ± 0.56	0.81 ± 0.64	2.00 ± 0.67
Concern about COVID-19 infection		*p*	<0.001***	0.007*	<0.001***	<0.001***
No	327 (54.4)		3.55 ± 0.42	0.90 ± 0.58	0.86 ± 0.67	2.08 ± 0.69
Yes	274 (45.6)		3.14 ± 0.51	0.77 ± 0.52	0.67 ± 0.61	1.82 ± 0.60

*p < 0.05, **p < 0.005, and ***p < 0.001, the test results are presented at point 3.3.

### 3.4 Analysis of influencing factors in COVID-19 panic, depression, anxiety, and PTSD

The current results showed that having a better maternal relationship was a protective factor for depression (OR = 0.5, 95% Cl: 0.28–0.91, *p* < 0.05), while a better paternal relationship was a protective factor for PTSD (OR = 0.45, 95% Cl: 0.21–0.97, *p* < 0.05). Suicidal thoughts were a risk factor for depression (OR = 5.01, 95% Cl: 1.73–14.53, *p* < 0.05), anxiety (OR = 5.98, 95% Cl: 2.63–13.6 *p* < 0.001) and PTSD (OR = 4.52, 95% Cl: 2.06–9.9, *p* < 0.001). Self-harm was a risk factor for COVID-19 panic (OR = 4.48, 95% Cl: 1.45–13.81, *p* < 0.05), depression (OR = 3.73, 95% Cl: 1.22–11.4, *p* < 0.05), anxiety (OR = 1.69, 95% Cl: 0.72–3.97, *p* < 0.05) and PTSD (OR = 3.72, 95% Cl: 1.78–7.83, *p* < 0.001). Additionally, close contact with COVID-19 patients was a risk factor for depression (OR = 2.92, 95% Cl: 1.03–8.29, *p* < 0.05), anxiety (OR = 2.27, 95% Cl: 1.05–4.92, *p* < 0.05), and PTSD (OR = 2.27, 95% Cl: 0.94–5.47, *p* < 0.05). This is similar to the study by Rassolnia and Nobari (2024), which pointed out that socioeconomic status and physical activity were important predictors of an individual’s mental health (Rassolnia and Nobari, 2024). Being in close contact with COVID-19 patients and concern about COVID-19 infection were protective factors for COVID-19 panic (OR = 0.2, 95% Cl: 0.11–0.35, *p* < 0.001; see Table 3 for details).

**Table 3:** Analysis of factors related to COVID-19 panic, depression, anxiety and PTSD at T4.

	COVID-19 panic OR [95%CI]	Depression OR [95%CI]	Anxiety OR [95%CI]	PTSD OR [95%CI]
Gender [Reference: Male]				
Female	1.29 [0.77–2.16]	1.27 [0.86–1.90]	1.05 [0.58–1.91]	0.83 [0.43–1.59]
Relationship with mother [Reference: General]				
Good	1.69 [0.82–3.48]	0.5** [0.28–0.91]	1.45 [0.64–3.28]	1.20 [0.53–2.72]
Relationship with father [Reference: General]				
Good	0.74 [0.38–1.43]	1.08 [0.67–1.74]	0.58 [0.29–1.16]	0.45* [0.21–0.97]
Suicidal thoughts [Reference: No]				
Yes	0.36* [0.17–0.79]	5.01** [1.73–14.53]	5.98*** [2.63–13.6]	4.52*** [2.06–9.9]
Self-harm [Reference: No]				
Yes	4.48* [1.45–13.81]	3.73* [1.22–11.4]	1.69* [0.72–3.97]	3.72*** [1.78–7.83]
Close contact with COVID-19 patients [Reference: No]				
Yes	0.36** [0.17–0.69]	2.92* [1.03–8.29]	2.27* [1.05–4.92]	2.27* [0.94–5.47]
Concern about COVID-19 infection [Reference: No]				
Yes	0.2*** [0.11–0.35]	0.79 [0.36–1.72]	0.46* [0.25–0.83]	1.89 [0.98–3.66]

*p < 0.05, **p < 0.005, and ***p < 0.001, the test results are presented at point 3.4.

### 3.5 Analysis of related factors of suicidal thoughts and self-harm

To better understand the relevant factors that influence the mental health of students, reports of suicidal thoughts and self-injury were investigated. Suicidal thoughts and self-harm were used as dependent variables, and COVID-19 panic, depression, anxiety, and PTSD were set as independent variables to conduct a binary logistical regression analysis. It was found that depression (OR = 3.19, 95% Cl: 1.04–9.75, *p* < 0.05) and PTSD (OR = 3.49, 95% Cl: 1.96–6.21, *p* < 0.001) were risk factors for suicidal thoughts. Meanwhile, COVID-19 panic (OR = 3.03, 95% Cl: 1.06–8.66, *p* < 0.05) and PTSD (OR = 2.26, 95% Cl: 1.3–3.93, *p* < 0.05) were risk factors for self-harming behavior (see Table 4 for details).

**Table 4:** Analysis of related factors of suicidal thoughts and self-harm.

	Suicidal thoughts OR (95%CI)	*p*	Self-harm OR (95%CI)	*p*
COVID-19 panic	0.59 (0.29–1.18)	0.137	3.03 (1.06–8.66)	0.038*
Depression	3.19 (1.04–9.75)	0.042*	1.87 (0.78–4.48)	0.158
Anxiety	1.63 (0.81–3.30)	0.174	1.64 (0.84–3.21)	0.146
PTSD	3.49 (1.96–6.21)	<0.001***	2.26 (1.3–3.93)	0.004**

*p < 0.05, **p < 0.005, and ***p < 0.001, the test results are presented at point 3.5.

## 4 Discussion

To the best of our knowledge, this is the first study examining the long-term psychological effects of the COVID-19 pandemic in college students following the full removal of restrictions. The current results highlight the importance of continuing to care for the mental health of college students after the pandemic. A GAD-7 scale threshold above 10 (i.e., moderate anxiety and above) is generally considered clinically significant. Four cross-sectional surveys revealed self-reported anxiety rates of 18, 19.9, 24.3, and 13.6%, respectively. The four-time self-reported prevalence of PTSD was 21.5, 20.9, 19.3, and 29.6%. The prevalence of anxiety observed in the current surveys is consistent with, or higher than, those reported in the majority of previous studies (Li et al., 2022; Qin et al., 2023). However, the rates observed were lower than the rates reported in an American study (Grineski et al., 2021). The study found that the prevalence of PTSD was much higher than reported in the majority of previous studies (Fu et al., 2013; Wang et al., 2022); however, this finding is consistent with those reported by Chen et al. (2023). Differences in prevalence rates may be related to the medical study location, scoring threshold, measurement tools, and/or environmental differences.

The prevalence of depression and anxiety in the four surveys in this study was higher than the pre-pandemic average among college students. For example, Jenkins et al. found an 18% prevalence of depression among college students before the pandemic (Jenkins et al., 2021). Ramon-Arbues et al. found that the prevalence of anxiety in college students before the pandemic was 23.6% (Ramón-Arbués et al., 2020). In addition, the prevalence of PTSD in the first two surveys was lower than that of Canadian scholars during SARS, and the latter two surveys were generally consistent with their findings, which found a 28.9% prevalence of PTSD (Hawryluck et al., 2004).

Our study found that within 2 months of full removal of COVID-19 restrictions, the infection rate among college students increased rapidly to 77.4%. This rate is slightly lower than the 80 to 90% infection rate predicted by the Chinese Center for Disease Control and Prevention, which may be due to the higher education level of college students. Specifically, their understanding of the epidemic may be higher than that of other groups, and they may be more willing to actively take protective measures, such as wearing a mask, voluntarily getting vaccinated, and avoiding crowded places. Recent findings from Taheri et al. (2023a) also suggest that elite athletes are more likely to have better mental health than non-elite athletes (Taheri et al., 2023b). The combined prevalence of anxiety decreased over time, and the results were consistent with those reported Bults et al. and during the SARS epidemic (Bults et al., 2011; Liu et al., 2021). Throughout the four surveys, the degree of COVID-19 fear among college students gradually decreased, but there was still a high proportion, which indicates that the COVID-19 pandemic has had a lasting impact on students. The likely reason for this is that most college students in China have never experienced such a large-scale public health event, as it has been 21 years since the last SARS outbreak. We suggest that schools and education departments should make long-term psychological plans to maintain the mental health of their students. In addition, we found no decrease in the rates of PTSD and suicidal thoughts. Since the removal of COVID-19 restrictions in China, a vast majority of college students have contracted the virus. However, PTSD has a long incubation period, up to 6 months or more, and many students may not develop PTSD in the early stages following the removal of restrictions (Merians et al., 2023). Po-Han and Emre’s study also found that PTSD was associated with an increased risk of suicidal thoughts (Chou et al., 2020; Umucu et al., 2022). Therefore, the continuous monitoring of these indicators is particularly important for college students.

In addition, this study found that students who had close contact with COVID-19 patients were more likely to develop depression, anxiety, and PTSD. There are several possible explanations for this result. First, during the pandemic, people who had close contact with COVID-19 positive patients were isolated for 14 days or more, which likely had a profound negative effect on the mental health of students. Second, COVID-19 is highly contagious and often presents with severe clinical symptoms. Compared with students who did not report contact with COVID-19 positive patients, the panic over COVID-19 may have increased the psychological burden on these individuals. Liu et al. also found that the greater the fear of COVID-19, the higher the PTSD symptom score (Liu et al., 2023). Interestingly, the current study found that maternal and paternal closeness were protective factors for depression and PTSD, respectively. This result is consistent with that obtained in the research of Tan et al. (2022). This may be due to the different parenting styles of mothers and fathers in China. In the vast majority of families in China, mothers spend more time with their children, and those children are more likely to receive love and care. Fathers, however, tend to be more resilient to stress, a quality that may subtly influence their children (Li and Guo, 2023).

Our study found that students with suicidal thoughts and self-harming behaviors were more likely to develop depression, anxiety, and PTSD. This result is consistent with a study conducted in Spain, which found that 74.1% of suicidal thoughts could be related to mental disorders and adverse life experiences associated with the pandemic (Mortier et al., 2021). Furthermore, numerous studies have shown that individuals with suicidal thoughts and self-injurious behaviors are more vulnerable (Iskric et al., 2020). Moreover, suicidal thoughts and self-harm behavior are often accompanied by some form of mental dysfunction (Kothgassner et al., 2021). In the fourth survey, we found a higher percentage of students with suicidal thoughts. This study found that students with PTSD were more likely to have suicidal thoughts and exhibit self-injurious behavior. Depressed students were also at risk for suicidal thoughts. Suicidal thoughts and self-harm continue to be major mental health problems among adolescents around the world (Kothgassner et al., 2021). Suicide is the leading cause of death among female adolescents in the Western world and the third leading cause of death among male adolescents (Kyu et al., 2016). Carmassi et al. surveyed survivors of the 2009 L ‘Aquila earthquake and found that PTSD was significantly associated with an increase in suicidal thoughts (Carmassi et al., 2021). Grossberg and Rice et al. also found that depression is an important risk factor for adolescent suicide (Grossberg and Rice, 2023).

It is worth mentioning that the current study found that students with COVID-19 panic were at risk for self-harm. A study in the UK found that nearly half of self-harming patients presenting to hospitals in the period after the lockdown were affected by COVID-related factors (Hawton et al., 2021). Similarly, a study by Paul et al. in the United Kingdom found that self-harm risk and behavior may have increased for a large proportion of adults during a pandemic (Paul and Fancourt, 2022). These students were all victims during the pandemic, and the experience of adverse events made them more likely to harm themselves.

Given that the mental health of college students after COVID-19 restrictions had been removed was still suboptimal, some proposals need to be put forward in combination with the findings of this study. First, intervention measures should aim at improving the psychological quality of college students. The psychological impact of the pandemic on college students was unprecedented, especially at the beginning of the pandemic and in the first few months after the removal of restrictions. Therefore, we recommend that mental-health curricula be strengthened, especially by adding or strengthening the post-disaster mental recovery component and that ongoing mental-health programs be developed. In addition, as mentioned in previous research, programs for fostering psychological capital (Yang and Yang, 2022) such as programs to foster empathy skills (Kroshus et al., 2021), programs to support psychological development (Arslan, 2022), and programs to promote communication and teamwork (Lochner et al., 2020) may be beneficial. Second, preventive measures should be aimed at improving students’ personal skills—that is, their ability to respond to disasters. Students’ fears may stem from not knowing what to do in the face of a disaster. Therefore, schools should focus on improving students’ ability to cope with disasters, including by popularizing relevant knowledge and imparting first-aid skills, which will prepare students for the next public health emergency. Third, the perspective of interventions should be expanded, such as by strengthening public health systems, providing necessary emergency resource protection, and having sufficient resources to protect students when public health emergencies occur.

In addition, we suggest that future research should continue to monitor the recovery of post-pandemic mental health among college students, especially the symptoms of PTSD, as PTSD typically has a long incubation period. Secondly, future research can develop more mental health measures and validate them, which will provide a broader reference for relevant departments. Third, the implementation of long-term longitudinal studies and multi-center studies will help to better understand the overall picture of college students’ mental health in the post-pandemic era.

### 4.1 Advantages and limitations

In this study, a small number of students participated in all four surveys, making it a quasi-longitudinal study. According to Steel (2008), while samples collected over different periods may not overlap, valid inferences can be made about overall worthwhile changes based on repeated cross-sectional designs (Steel, 2008). Repeated cross-sectional designs can overcome the difficulties of longitudinal data collection, especially in the special context of a pandemic, where a high degree of overlap between samples may not be necessary for trends in college students’ mental health over time (González-Nuevo et al., 2024). However, the limitations of this study need to be pointed out to improve subsequent research. First, our study was not a multicenter study, limiting the generalizability of the findings; future research will expand the scope of study participants. Second, for unavoidable reasons, the data collected in the fourth survey of this study were transferred from paper questionnaires to network questionnaires. Although we tried our best to control the data deviations caused by different collection methods, the comparability of data was still likely affected. Future studies should maintain consistency in collection methods when collecting longitudinal data.

## 5 Conclusion

This is the first study to examine the trajectory of college students’ mental health over time. We found showed that a year after the COVID-19 pandemic, the overall mental health of college students was not optimal. Hence, we found showed that the long-term impact of COVID-19 on the mental health of college students may have already accumulated. To mitigate this impact and prepare for the next major public health event, strengthening college students’ mental health curricula and promoting healthy behaviors among college students should be a priority for universities and education authorities.

## Data availability statement

The raw data supporting the conclusions of this article will be made available by the authors, without undue reservation.

## Ethics statement

The studies involving humans were approved by the Ethics Committee of North Sichuan Medical College (ID No. 2023004). The studies were conducted in accordance with the local legislation and institutional requirements. The participants provided their written informed consent to participate in this study. Written informed consent was obtained from the individual(s) for the publication of any potentially identifiable images or data included in this article.

## Author contributions

YC: Data curation, Formal analysis, Investigation, Methodology, Software, Visualization, Writing – original draft. XK: Conceptualization, Funding acquisition, Methodology, Resources, Supervision, Validation, Writing – review & editing. JL: Investigation, Methodology, Writing – original draft. JD: Writing – review & editing. JZ: Investigation, Writing – original draft. XJ: Investigation, Writing – original draft. TZ: Investigation, Writing – original draft. XX: Writing – original draft.
